# An Account of Diseases in an Eastern District of London, from June 20 to July 20, 1810

**Published:** 1810-08

**Authors:** 


### *An Account of Diseases in an Eastern District of London, from June 20 to July 20, 1810.*

| | |
|---|---|
| ACUTE DISEASES. | |
| Peripneumonia Notha | 3 |
| Cynanche Tonsillaris | 5 |
| Dysenteria - - - - - | 2 |
| Rheumatismus Acutus - | 3 |
| CHRONIC DISEASES. | |
| Tussis - - - - - - - - | 10 |
| Dyspnœa - - - - - | 5 |
| Tussis cum Dyspnœa - | 4 |
| Hæmoptysis - - - - - | 5 |
| Phthisis Pulmonalis - - | 3 |
| Catarrhus - - - - - - | 4 |
| Pleurodyne - - - - - | 3 |
| Anasarca - - - - - - | 2 |
| Ascites - - - - - - - | 1 |
| Enterodynia - - - - - | 4 |
| Menorrhagia - - - - - | 5 |
| Fluor Albus - - - - - | 7 |
| Amenorrhœa - - - - - | 3 |
| Procidentia Ani - - - - | 1 |
| Epilepsia - - - - - - | 1 |
| Urticaria - - - - - - - | 2 |
| Rheumatismus Chronicus | 12 |
| PUERPERAL DISEASES. | |
| Ephemera - - - - - - | 3 |
| Menorrhagia Lochialis - | 5 |
| Mastodynia - - - - - | 6 |
| Rhagas Papillæ - - - - | 3 |
| INFANTILE DISEASES. | |
| Febris Mesenterica - - | 2 |
| Erysipelas Infantile - - | 1 |
| Vermes - - - - - - - - | 4 |
| Convulsio - - - - - - | 3 |
| Pertussis - - - - - - | 6 |

During the last few weeks a considerable change has taken place in the state of the weather. After a very long continuance of Easterly and North Easterly winds, and an almost total absence of rain, in consequence of which, the surface of the earth assumed a most dry and sterile appearance, the occasional changes of the wind to the West and South West, together with some very copious showers of rain, have produced a favourable appearance, and given the hope of a more plentiful supply of the necessary articles of food both for man and beast. This alteration has also proved favourable to the human constitution, and some diseases which were aggravated by the long continuance of the cold winds have now assumed a milder form. Still, however, the very quick alternation of heat and cold has exposed many persons, especially those of a delicate habit, to considerable inconvenience. The difference of temperature, rature, even at the same hour, under the influence of the sun's ray, and in a situation exposed to the Eastern winds, which have still in some degree prevailed, has produced unpleasant effects. Pains of the face and teeth have been very common. These might be in some instances traced to a carious tooth, the removal of which has proved a radical cure; but in other cases they have appeared to be a symptom of rheumatism. This disease, as it is well known, appears in such a variety of forms, and is so connected with some peculiarities of constitution, that it is sometimes not easy to detect it, and the cure is at all times tedious and difficult. Affections of the throat have been very frequent during the period referred to. Cynanche tonsillaris has appeared in several instances, but in a milder form than it sometimes assumes. It has been rather a local than general disease; even where the tonsils have been considerably enlarged, and where difficulty and pain of deglutition have been felt, the degree of fever has been inconsiderable. The use of a domestic gargle, and the opening of the bowels with small doses of cathartic salts, have been the only means that were found necessary to the cure.

